# Empirically testing vaterite structural models using neutron diffraction and thermal analysis

**DOI:** 10.1038/srep36799

**Published:** 2016-11-18

**Authors:** Bryan C. Chakoumakos, Brenda M. Pracheil, Ryan P. Koenigs, Ronald M. Bruch, Mikhail Feygenson

**Affiliations:** 1 Oak Ridge National Laboratory, Quantum Condensed Matter Division, Oak Ridge, TN 37831, USA; 2 Oak Ridge National Laboratory, Environmental Sciences Division, Oak Ridge, TN 37831, USA; 3 Wisconsin Department of Natural Resources, Oshkosh, Wisconsin, 54901, USA

## Abstract

Otoliths, calcium carbonate (CaCO_3_) ear bones, are among the most commonly used age and growth structures of fishes. Most fish otoliths are comprised of the most dense CaCO_3_ polymorph, aragonite. Sturgeon otoliths, in contrast, have been characterized as the rare and structurally enigmatic polymorph, vaterite—a metastable polymorph of CaCO_3_. Vaterite is an important material ranging from biomedical to personal care applications although its crystal structure is highly debated. We characterized the structure of Lake Sturgeon otoliths using thermal analysis and neutron powder diffraction, which is used non-destructively. We confirmed that while Lake Sturgeon otoliths are primarily composed of vaterite, they also contain the denser CaCO_3_ polymorph, calcite. For the vaterite fraction, neutron diffraction data provide enhanced discrimination of the carbonate group compared to x-ray diffraction data, owing to the different relative neutron scattering lengths, and thus offer the opportunity to uniquely test the more than one dozen crystal structural models that have been proposed for vaterite. Of those, space group *P*6_5_22 model, a = 7.1443(4)Å, c = 25.350(4)Å, V = 1121.5(2)Å^3^ provides the best fit to the neutron powder diffraction data, and allows for a structure refinement using rigid carbonate groups.

Vaterite, a metastable polymorph of calcium carbonate (CaCO_3_)^1^ is of substantial interest as a naturally occurring biomaterial, and for its use as an additive in various consumer products ranging from paper and coatings to plastic and elastomer reinforcement to food supplements, oral hygiene aids, and cosmetics^2^. For instance, vaterite’s greater ability to dissolve in body fluids compared to other polymorphs of CaCO_3_ makes it desirable for manufacturing nanocapsules for delivering drugs at the cellular level^3^. Despite the realized and potential importance of vaterite to humans, some of the most basic facts about this compound such as its crystalline structure have challenged researchers. Currently, more than a dozen crystal structure models have been proposed with little weight favoring one over the others^1^.

Many basic properties of vaterite are unresolved due in part to the rarity of naturally occurring vaterite. Naturally occurring vaterite structures include human gallstones and portions of fish ear bones, or otoliths, from primitive fishes such as sturgeons^4^ or sporadically from more modern fishes such as salmon and trout, and are potentially formed in response to physiological stress, especially thermal stress^5^ and captive rearing^6^,^7^. In fact, only a few reliable sources of biogenic vaterite exist for study including sea squirt spicules^8^,^9^ and otoliths of sturgeons, but only rarely those of other fish or animals. The ability of making biogenic vaterite has apparently been retained through evolution in some fish such as salmonids because these fishes sometimes have vaterite patches in their otoliths^4^,^10^, but sturgeons are really the sole reliable source of vaterite from fish otoliths.

Unlike calcite and aragonite whose crystal structures were easily established early on by diffraction methods, the exact nature of the vaterite crystal structure has been the subject of ongoing and inconclusive debate. This is due in part to the microcrystalline nature of most synthetic and natural forms which limits the solution methodologies, but moreover, to the similarities between a variety of partially disordered and ordered structural models that can provide seemingly good fits to the diffraction data using x-rays or electrons. Kabalah-Amitai *et al*.^9^ concluded that vaterite from sea squirt spicules is actually composed of at least two different crystallographic structures that coexist within a pseudo–single crystal, based on their electron diffraction study. The various structural models proposed for vaterite are based on unit cells indexed from single-crystal x-ray diffraction, powder lab-based and synchrotron x-ray diffraction, and electron diffraction. An even greater number of theoretically calculated structures have also been proposed; see Table S1. This history has recently been reviewed by Wang *et al*.^11^ and Burgess and Bryce^12^, who also summarize the structural constraints imposed by complementary techniques, specifically vibrational (infrared and Raman) and NMR spectroscopies. More recently, many of the proposed structural models for vaterite have been interpreted within the order-disorder (OD) theory which systematizes them as polytypic stackings of a common layer module^13^. Table S1 summarizes the various proposed models and in Fig. 1 we compare the normalized volumes and the densities of these models with those of calcite and aragonite. Vaterite transforms irreversibly to calcite in the temperature range 420–515 °C; the volume change is widely accepted to be negative, although the equilibrium phase relations of vaterite with respect to calcite and aragonite have not been well established^14^,^15^. Nevertheless, we expect that the density of vaterite is less than that of calcite, given the preponderance of experimental measurements. On this basis, those models proposed with densities the same as, or greater than, calcite (2.72 g/cm^3^) seem unlikely candidates for vaterite.

To our knowledge, neutron diffraction has never been used to study vaterite, yet it offers several advantages that suggest that this technique may provide empirical weight to one or more of the list of proposed structural models. For instance, prior studies have used X-ray and electron diffraction, but to its advantage, neutron diffraction is a bulk probe, meaning diffraction patterns of the entire otolith (even for cm sized ones) can be recorded without any sample preparation. In addition, neutron diffraction data provide somewhat enhanced discrimination of the carbonate group compared to x-ray or electron diffraction data, owing to the different relative neutron scattering lengths of the constituent elements. These properties of neutron diffraction position it to provide a truly unique test of the more than one dozen crystal structural models that have been proposed for vaterite. In addition, establishing a protocol for examining whole intact otoliths by neutron diffraction can realize non-destructive quantification of their individual phase fractions when more than one CaCO_3_ polymorph is present.

## Results

**Figure 2:**
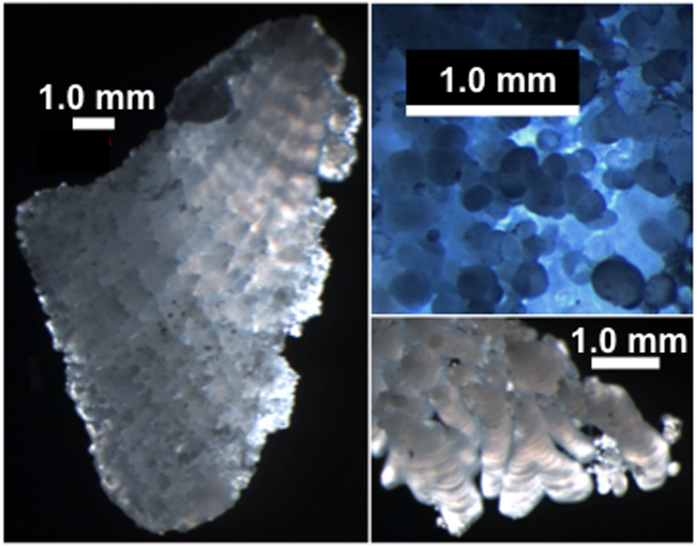
Lake Sturgeon otoliths comprised of mostly vaterite + minor calcite show globular accumulation of calcium carbonate phases on their exterior, sometimes porous and irregular margins, and banded growth structures at different length scales.

A typical Lake Sturgeon (*Acipenser fulvescens*) otolith is shown in Fig. 2. Most of the exterior consists of microcrystalline globules 1–100 *μ*m in size. The total mass of a typical otolith is 100–200 mg. Preliminary x-ray and neutron powder diffraction showed the presence of two carbonate phases, vaterite and calcite, in both the powdered and whole Lake Sturgeon otoliths.

**Figure 3:**
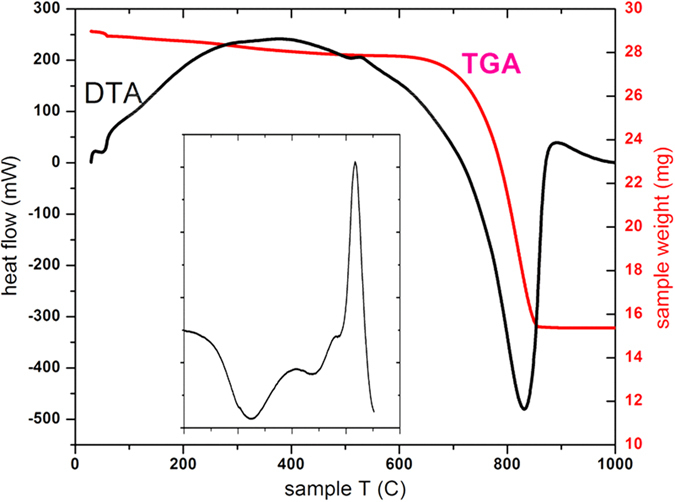
Thermal analysis of powdered Lake Sturgeon otoliths, composed of vaterite + calcite. Major weight loss in the TGA above 700 °C is decarbonation during decomposition of the carbonates, and corresponds to the major endotherm in the DTA. Inset: The tiny exotherm near 515 °C, attributed to the transformation of vaterite to calcite in the DTA, is better shown by DSC, and the weaker endotherms at 325 °C and 440 °C are likely due to dehydrations. In this case, the baseline heat-flow from a second heating of the sample was subtracted. The second heating showed no peaks and varied smoothly, consistent with the transition of vaterite to calcite being irreversible.

The combination of TGA, DTA, and DSC on two different powdered samples and one whole otolith fragment each showed the same behavior, a small exotherm at 515 °C due to the transformation of the vaterite phase fraction to calcite and a large endotherm above 700 °C due to the decomposition (decarbonation) of the calcite (Fig. 3). The accompanying weight loss in the TGA is consistent with the loss of CO_2_ from the carbonate decomposition, and provides an independent confirmation that the otolith is comprised almost solely of calcium carbonate phases. Two small endotherms at 325 °C and 440 °C in the DSC (Fig. 3, inset) are likely due to the loss of physisorbed waters (3 wt percent from the TGA), although no chemical analysis of the exhaust gas was made during the thermal analysis. The 515 °C temperature for the vaterite to calcite transformation we find is somewhat higher compared to other reports (e.g., 377–477 °C^15^; 420–477 °C^16^) for various synthetic and natural product samples, but the transformation temperature is known to be dependent on preparation method for synthetic samples and origin for natural materials. It has been clearly shown that the transformation temperature shifts higher for increasing heating rate, and the presence of calcite or aragonite either slightly accelerates or retards the transformation, respectively^17^.

Initially, we considered the previously proposed structural models that were less dense than calcite and which had the smallest unit cell volumes. These include the originally proposed hexagonal and orthorhombic models^18^,^19^,^20^. None of these fit well, so we went directly to test the carbonate ordered superstructure model (*P*6_5_22)^11^ shown as the best fit of synchrotron x-ray powder data for a synthetic sample^21^. They had tested 5 other structural models, *P*6_3_/*mmc*, *P*3_2_21, *Pnma*, *Ama*2^20^, *C*2/*c*; however, for those the Rietveld fits were not as good. Similarly, we found an improved fit using the *P*6_5_22 model for our neutron powder diffraction data.

**Figure 4:**
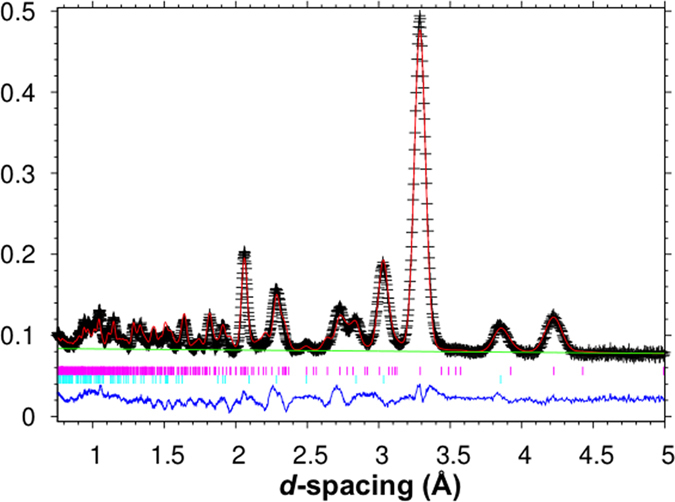
Example Rietveld refinement fit for Lake Sturgeon otoliths from detector 31° bank of the NOMAD neutron powder diffractometer. Crosses are observed data and the solid black line is the best fit. The green solid line is the fixed linear background. The reflection markers are vaterite (upper magenta) and calcite (lower turquoise). The difference curve between the model and the observed pattern is shown in blue at the bottom of the panel.

For the Rietveld refinements, backgrounds were refined linear fits. Although, unconstrained refinements of all of the structural parameters of both phases can be included in the least squares fitting, the refined geometries of the carbonate groups deviate significantly from their typically expected bond lengths and angles for the vaterite phase. Therefore, rigid bodies (RB) for the carbonate groups in the vaterite phase were introduced, which reduce the number of structural parameters from 29 (unconstrained isotropic model) to 17 (RB isotropic model) for the vaterite phase. Because all of the oxygen atoms belong to carbonate groups, the RB model is tantamount to packing rigid carbonate groups with calcium atoms. The carbonate groups were allowed to change in size, to move as allowed by the site symmetry, and to displace isotropically about their mean position. Due to the symmetry constraints in the *P*6_5_22 space group model, the one carbonate group RB (C2) is only allowed to translate along the x-direction and rotate around the C-O1 bond, whereas the other carbonate group RB (C1) is free to translate in all three directions as well as rotate around three orthogonal directions. The refined structural parameters for the calcite phase agree reasonably well with high quality structural refinements previously reported for calcite, so in this sense, the overall quality of the two-phase refinement seems to be free of systematic error. Table 1 presents the refined structural parameters for a powdered Lake Sturgeon otolith. For the calcite phase a March–Dollase preferred orientation parameter was included; however, for the vaterite phase preferred orientation was not apparent. The quantitative analysis of the phase fractions of vaterite and calcite for the powdered sample, which represents a sampling of more than one otolith, is 76.5(1) wt. percent vaterite +23.5(1) wt. percent calcite. Fig. 4 shows an example Rietveld refinement fit for the neutron powder diffraction data. Additional fits to other detector banks are in the Supplemental Material. The phase fractions of several whole otoliths, not surprisingly, had the same average composition, but individually varied over the range 65–84 wt. percent vaterite. Crystallographic Information Files have been deposited in the Inorganic Crystal Structure Database and are available in the Supplementary Materials.

## Discussion

**Figure 5:**
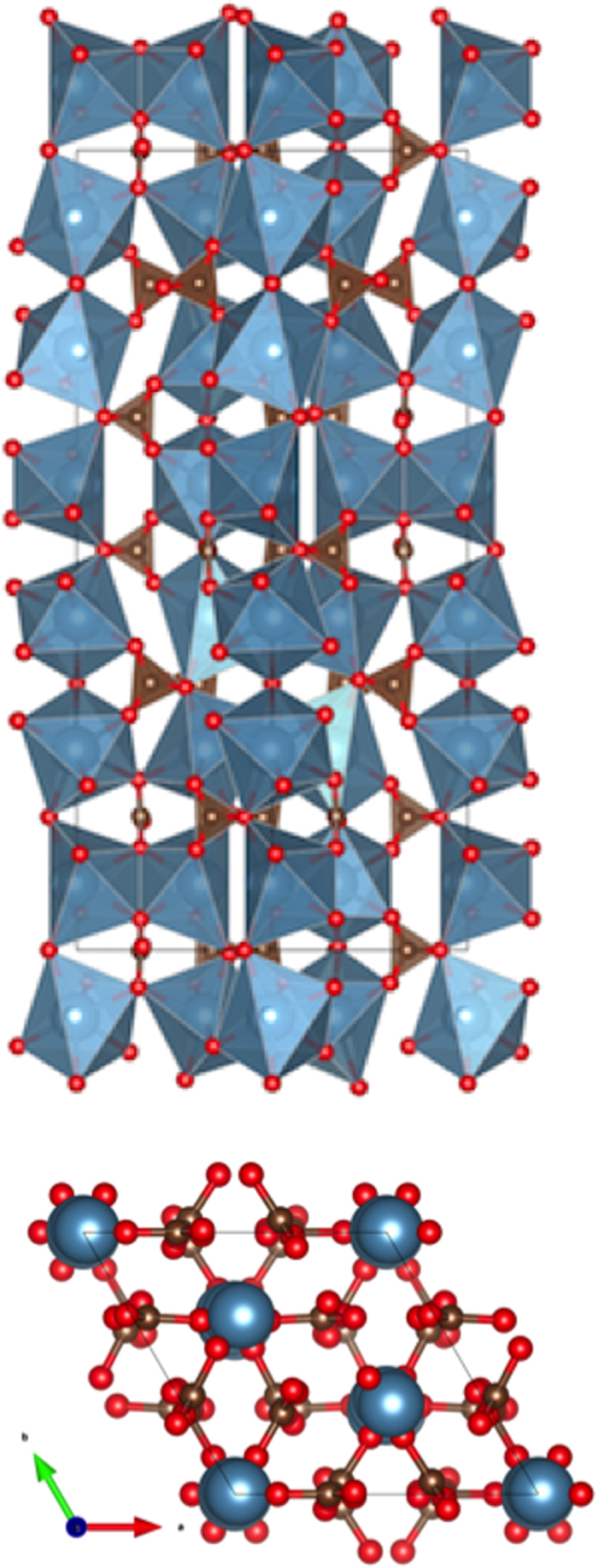
Upper: Polyhedral representation of the vaterite crystal structure (space group *P*6_5_22) projected along (110) refined from neutron powder diffraction data. Blue-shaded polyhedral are calcium oxide octahedra, and the brown shaded triangles are carbonate groups. Of the three unique calcium oxide octahedra Ca1 and Ca2 share an edge. All other polyhedral linkages are by corner-sharing. The unit cell is outlined. Lower: (001) projection, blue balls = Ca atoms, grey balls = C atoms, red balls = oxygen atoms.

The vaterite crystal structure (Fig. 5) is less dense than calcite as expected, but it has one feature in common with aragonite in having a shared edge between two of the three CaO_6_ octahedra. These two octahedra are quite distorted. All other linkages between the CaO_6_ octahedra and the carbonate groups are corner-sharing as is the case for calcite. The Ca1 and Ca2 that share a polyhedral edge are both off-centered in their oxide octahedra to maintain an optimal Ca…Ca distance of 4.04 Å, compared to 4.085 Å in calcite. The near-neighbor C…C distances in this vaterite model are intermediate between those of calcite and aragonite (Table S2). The space group *P*6_5_22 of this vaterite model is chiral, and necessarily acentric, so the notion that biological molecules with specific handedness could template nucleation cannot be ruled out.

**Figure 6:**
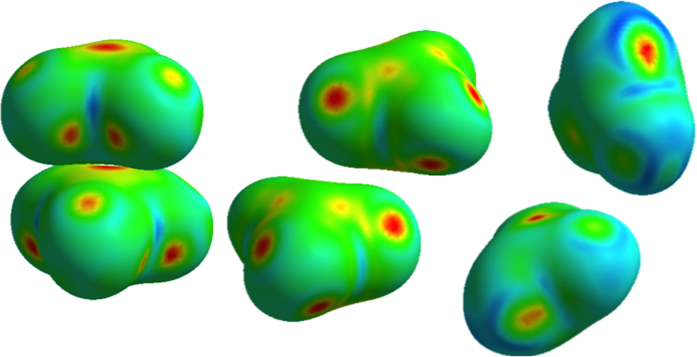
Hirshfeld surfaces of neighboring equivalent carbonate groups in aragonite (left), calcite (center) and the inequivalent carbonate groups in vaterite (right), each contoured by curvedness. Flatter parts are indicated by hotter colors and correlate with increasing bonding interaction.

If one examines the Hirshfeld surfaces^22^ of the carbonates groups in vaterite, calcite and aragonite generated using CrystalExplorer^23^, a picture emerges that emphasizes the interactions between the carbonate groups as a contributor to the stability of each of the calcium carbonate polymorphs (Fig. 6). The Hirshfeld surface is the isosurface level that partitions half of the electron density between the carbonate group and the remaining unit cell. The Hirshfeld surface effectively partitions the crystal space into molecular and/or atomic units, and the inter-molecular (atomic) interactions correlate with the flat areas of the curvedness contoured on the Hirshfeld surface. Given this notion, distinct differences between the carbonate group interactions in the three polymorphs can be seen. In aragonite, the densest form, the carbonate groups are stacked with a strong interaction indicated center-to-center, and C…C distance of 2.88 Å. In calcite, the interactions are weak between the carbonate groups, and the C…C is 4.04 Å. In vaterite, the interactions between carbonate groups are both weak and strong; nearest neighbor C…C distances are between 3.88 and 4.78 Å. Crystal structure projections of all three polymorphs are presented in Fig. S2.

The microstructure of the calcite + vaterite assemblage in these otoliths has not been explored yet, but it is amenable to study by Raman spectroscopy and Electron Backscatter Diffraction (EBSD) if the individual crystals are of sufficient size. In principle these additional complementary methods can provide individual grain orientations, and local texture, on the surfaces of cut and polished otolith sections.

In summary, neutron diffraction offers a novel, non-destructive measure of the phase fractions in fish otoliths. Lake Sturgeon otoliths consisting of the major phase vaterite + a minority phase calcite, allow proposed structural models for vaterite to be uniquely tested by Rietveld refinement methods. The hexagonal structural model (space group *P*6_5_22)^21^ provides the best fit to our neutron data, and is the same model that also provided the best fit to synchrotron X-ray diffraction data^11^. The neutron data allow for refinement of the carbonate group and calcium atom positions and their displacement parameters using the rigid-body formalism for the carbonate groups. The near-neighbor carbonate group arrangements in calcite, aragonite and vaterite suggest that the relative stability of these polymorphs is determined by the carbonate group interactions.

## Methods

Otoliths were obtained from legally harvested Lake Sturgeon (*Acipenser fulvescens*) caught by anglers on Lake Winnebago, Wisconsin. Experiments described in this study were not conducted on live vertebrates, rather, sturgeon otoliths used in this study were voluntarily contributed by state-licensed anglers in a sustainably-managed Lake Sturgeon sport fishery. No live animals were handled by the authors for the purposes of this study, therefore, institutional animal use and care regulations did not apply. Worldwide, most sturgeons are either threatened or endangered, however, the Lake Winnebago sturgeon comprise a sustainable fishery that has been carefully managed for more than a century^24^.

Because we were interested in material properties of vaterite, which should be invariant among individual sturgeon, we initially examined powdered samples consisting of otoliths from several individuals, and subsequently examined several whole intact otoliths to quantify individual variations in their phase fractions. It is possible that the carbonate crystals within an otolith have a preferred orientation, that is, a preferred direction of growth relative to the overall shape of the otolith. Preferred orientation can be modeled, but reducing or avoiding it altogether is desirable, otherwise altered intensity distributions in multiphase samples can confound interpretations. To reduce preferred orientation from the otolith material, we powdered several otoliths with a mortar and pestle and analyzed their crystalline structure using neutron powder diffraction. Powdered otoliths were exposed to the neutron beam by placing approximately 50 mg of powder into 3 mm diameter glass capillaries. We also suspended three whole otoliths individually in the neutron beam. For both powder and whole otolith neutron diffraction, typical sample masses ranged from 50–150 mg.

Neutron diffraction was carried out using the time-of-flight Nanoscale-Ordered Materials Diffractometer (NOMAD) at the Spallation Neutron Source, Oak Ridge National Laboratory. NOMAD is a high-flux, medium-resolution diffractometer that uses a large bandwidth of neutron energies and six different detector banks, which afford a broad range of scattering angles (Q-range) and peak resolutions (*δ*Q/Q). NOMAD offers high intensity to provide short counting times (mins) and sufficient resolution to resolve these generally high symmetry, small unit cell structures (calcite, aragonite, vaterite). Data collection times for the otolith samples were typically 1 hr, and 1 hr for background measurements of an empty capillary or empty instrument that were subtracted from the raw data.

Rietveld refinements of the neutron powder diffraction data were made using GSAS^25^,^26^,^27^. Typically, we used a combination of the intermediate resolution detector banks, 2 (31°), 3 (67°), and 4 (122°) on the NOMAD instrument. Both calcite and vaterite were included in the fitting model. Hirshfeld surfaces^22^ of the carbonates groups in vaterite, calcite and aragonite were generated using CrystalExplorer^23^ to compare and contrast the differences in interactions between the carbonate groups in the three polymorphs.

X-ray powder diffraction, thermal analysis, and optical microscopy were also used as characterization methods. Simultaneous thermogravimetric analysis (TGA) and differential thermal analysis (DTA) were made at a heating rate of 10 °C/min using a Perkin Elmer Diamond TG/DTA. Differential scanning calorimetry (DSC) was made at a heating rate of 20 °C/min using a Perkin Elmer Pyris-1 DSC. The thermal analysis was done to see if the phase transformation behavior of vaterite was consistent with other reports. Earlier, we showed that sample preparation by hand grinding does not transform vaterite to calcite^10^.

## Additional Information

**How to cite this article**: Chakoumakos, B. C. *et al*. Empirically testing vaterite structural models using neutron diffraction and thermal analysis. *Sci. Rep.* **6**, 36799; doi: 10.1038/srep36799 (2016).

**Publisher’s note:** Springer Nature remains neutral with regard to jurisdictional claims in published maps and institutional affiliations.

## Supplementary Material

Supplementary Information

## Figures and Tables

**Figure 1:**
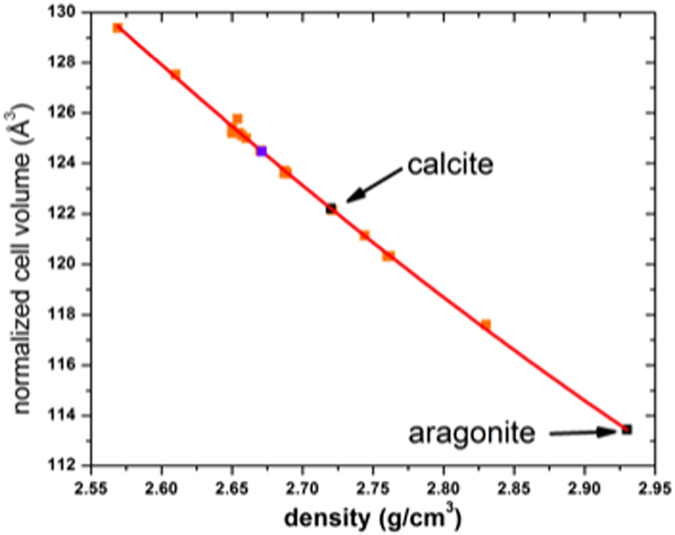
Density–volume plot for the various proposed structural models for vaterite (orange symbols) as compared to calcite and aragonite (black symbols). Our refined model for vaterite is shown by the purple symbol. The fitted curve is a 2nd order polynomial.

**Table 1:** Refined structural parameters for vaterite and calcite in Lake Sturgeon otoliths using neutron powder diffraction data from NOMAD 31°, 67°, and 122° detector banks (CIF available in supplementary materials).

Atom	Wyckoff site	Symmetry	*x*	*y*	*z*	*U*_*iso*_Å^2^
vaterite phase, space group *P*6_5_22 *a* = 7.1443(4), *c* = 25.350(4) Å, *V* = 1121.5(2)Å^3^						
Ca1	6*b*	2	0.007(2)	−*x*	1/12	0.009(3)*
Ca2	6*b*	2	0.681(2)	−x	1/12	0.009(3)*
Ca3	6*b*	2	0.343(2)	−*x*	1/12	0.014(8)
C2	6*a*	2	0.691(2)	0	1/2	0.027(4)
O1	6*a*	2	0.859(2)	0	1/2	0.027(4)
O2	12*c*	1	0.602(1)	−0.008(3)	0.5409(4)	0.027(4)
C1	12*c*	1	0.375(2)	0.327(2)	0.1658(3)	0.024(2)
O3	12*c*	1	0.556(2)	0.337(3)	0.1714(5)	0.024(2)
O4	12*c*	1	0.270(2)	0.326(3)	0.2059(4)	0.024(2)
O5	12*c*	1	0.300(2)	0.317(3)	0.1201(4)	0.024(2)
calcite phase, space group *R* 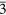 *c a* = 4.9856(6), *c* = 17.046(3)Å, *V* = 366.94(9) Å^3^						
Ca	6*b*	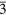	0	0	0	0.004(2)
C	6*a*	32	0	0	1/4	0.014(2)
O	18*e*	2	0.2607(8)	0	1/4	0.013(1)

Atoms C2, O1, O2 and C1, O3, O4, O5 constitute the rigid bodies C2 and C1, respectively.

^*^The isotropic *U*’s for Ca1 and Ca2 were constrained to be the same, otherwise the least squares would not converge.
