# Supplementary material for: Empirically testing vaterite structural models using neutron diffraction and thermal analysis
Source: Sci Rep. 2016 Nov 18;6:36799. doi: 10.1038/srep36799 (PMC5114672; doi:10.1038/srep36799)
Supplement: Supplementary Information [file srep36799-s1.pdf]

Supplementary Material

Empirically testing vaterite structural models using neutron diffraction and thermal analysis

Bryan C. Chakoumakos, Brenda M. Pracheil, Ryan P. Koenigs, Ronald M. Bruch, Mikhail Feygenson

Table S1. Vaterite crystal structural models

Space Group	Cell dimensions (Å, °)	density (g/cm^3)	notes	reference
$P6_3/mmc$	a = 4.13, b = 8.49	2.65	exp	4, 5
Pbnm	a = 4.531, b = 6.64, c = 8.477	2.63	theory	7
Pbnm	a = 4.341, b = 6.432, c = 8.424	2.83	theory	7
Pnma	a = 4.1291, b = 7.1581, c = 8.4764	2.655	exp	3, 5
$P6_522$	a = 7.29, c = 25.302	2.569	theory	2, 6
*Ama*2	a = 8.4721, b = 7.1575, c = 4.1265	2.657	exp	5, 10
*C*2/*c*	a = 12.17, b = 7.12, c = 9.47<br>β = 118.37	2.762	exp	8, 10
$C\bar{1}$	a = 12.17, b = 7.12, c = 25.32<br>α = 90., β = 99.22, γ = 90.	2.76	exp	8
$P3_221$	a = 7.1239, c = 25.3203	2.688	theory	9
*Ama*2	a = 8.4905, b = 6.3905, c = 4.5026	2.721	theory	9
$P2_12_12_1$	a = 4.3668, b = 6.5831, c = 8.4282	2.744	theory	9
$P6_5$	a = 7.112, c = 25.4089	2.688	theory	9
*C*1	a = 12.353, b = 7.102, c = 25.733<br>α = 90.46, β = 99.78, γ = 90.24	2.689	theory	1
*C*1	a = 12.358, b = 7.106, c = 25.741<br>α = 90.43, β = 99.88, γ = 90.29	2.687	theory	1
Cc	a = 12.245, b = 7.142, c = 9.371<br>β = 115.48	2.687	theory	1
*C*2	a = 12.281, b = 7.197, c = 9.305<br>β = 115.16	2.688	theory	1

(1) Demichelis, R.; Raiteri, P.; Gale, J. D.; Dovesi, R. The multiple structures of vaterite. *Cryst. Growth Des.* **2013**, *13*, 2247-2251.
(2) Wang J.; Zhang, F.; Zhang, J.; Ewing, R. C.; Becker, U.; Cai, Z. Carbonate orientational order and superlattice structure in vaterite. *J. Cryst. Growth* **2014**, *407*, 78-86.
(3) Meyer, H. J. Struktur und Fehlordnung des Vaterits. *Zeitschrift fur Kristallograhie* **1969**, *128,* 183-212 (in German with English abs.).
(4) Kamhi, S.R. On the structure of vaterite, $CaCO_3$. *Acta Crystallogr.* **1963**, *16,* 770-772.
(5) Le Bail, A.; Ouhenia, S.; Chateigner, D. Microtwinning hypothesis for a more ordered vaterite model. *Powder Diffr.* **2011**, *26*, 16-21.
(6) Wang, J.; Becker, U. Structure and carbonate orientation of vaterite ($CaCO_3$). *Am. Mineral.* **2009**, *94*, 380-386.
(7) Medeiros, S.; Albuquerque, E. L.; Maia, F. F.; Caetano, E. W. S.; Freire, V. N. First-principles calculations of structural, electronic, and optical absorption properties of $CaCO_3$ vaterite. *Chem. Phys. Lett.* **2007**, *435*, 59-64.
(8) Mugnaioli, E.; Adrusenko, I.; Schüler, T.; Loges, N.; Dinnebier, R. E.; Panthöfer, M.; Tremel, W.; Kolb, U. Ab initio structure determination of vaterite by automated electron diffraction. *Angew. Chem., Int. Ed.* **2012**, *51*, 7041–7045.
(9) Demichelis R.; Raiteri, P.; Gale, J. D.; Dovesi, R. A new structural model for disorder in vaterite from first-principles calculations. *CrystEngComm* **2012**, *14*, 44-47.
(10) Burgess, K.M.N. and Bryce, D.L. On the crystal structure of vaterite polymorph of CaCO3: A calcium-43 solid-state NMR and computational assessment. *Solid State Nucl. Mag. Res.* **2015**, *65*, 75-83.

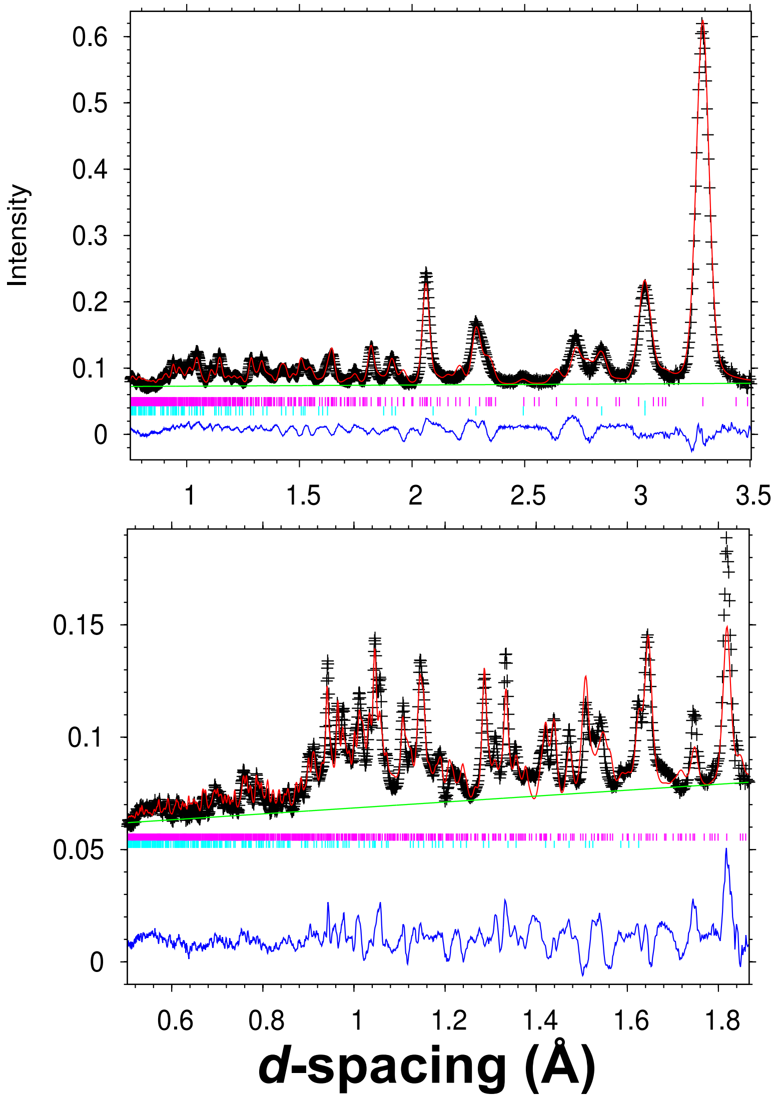

Fig. S1. Example Rietveld refinement fits for lake sturgeon otoliths from detector bank 3 and 4 of the NOMAD neutron powder diffractometer. Crosses are observed data and the solid black line is the best fit. The green solid line is the fixed linear background. The reflection markers are vaterite (upper magenta) and calcite (lower turquoise). The difference curve between the model and the observed pattern is shown in blue at the bottom of the panel.

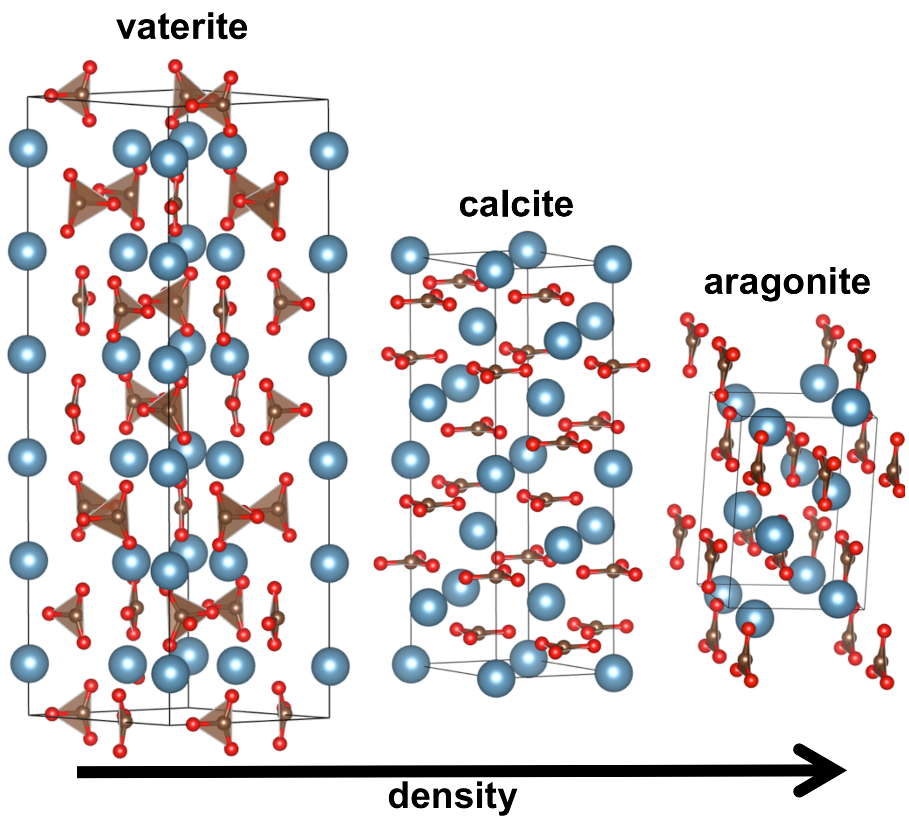

Fig. S2. Comparison of the crystal structures for vaterite, calcite and aragonite. Carbonate groups are shaded. Ca atoms are blue balls, oxygen atoms are red balls.

Table S2. Comparison of interatomic distances (Å) for vaterite, calcite, and aragonite

vaterite		calcite		calcite		aragonite	
this work		this work		Maslen et al. (1993)		Ye et al. (2012)	
Ca1-O1	2.308(9)	Ca1-O1	2.348(3)	Ca1-O1	2.3590(9)	Ca1-O2	2.5172(12)
Ca1-O4	2.510(13)	Ca1-O1	2.3479(5)	Ca1-O1	2.3590(5)	Ca1-O1	2.6527(7)
Ca1-O5	2.401(13)	Ca1-O1	2.3479(9)	Ca1-O1	2.3590(4)	Ca1-O2	2.4498(16)
Ca1-O4	2.510(19)	Ca1-O1	2.3479(9)	Ca1-O1	2.3590(4)	Ca1-O1	2.4165(18)
Ca1-O1	2.308(4)	Ca1-O1	2.348(3)	Ca1-O1	2.3590(9)	Ca1-O2	2.5467(11)
Ca1-O5	2.401(16)	Ca1-O1	2.3479(5)	Ca1-O1	2.3590(5)	Ca1-O2	2.5172(12)
						Ca1-O2	2.4498(16)
						Ca1-O2	2.5467(11)
						Ca1-O1	2.6527(7)
mean	2.406		2.348		2.359		2.527
Ca2-O2	2.384(17)						
Ca2-O3	2.432(13)						
Ca2-O4	2.35(2)						
Ca2-O3	2.432(15)						
Ca2-O4	2.355(14)						
Ca2-O2	2.384(14)						
mean	2.389						
Ca3-O2	2.58(2)						
Ca3-O2	2.583(19)						
Ca3-O3	2.154(14)						
Ca3-O3	2.154(13)						
Ca3-O5	2.47(2) Å						
Ca3-O5	2.468(17)						
mean	2.401						
C1-O3	1.264(15)	C1-O1	1.300(4)	C1-O1	1.2842(12)	C1-O1	1.274(3)
C1-O5	1.265(14)	C1-O1	1.2997(3)	C1-O1	1.2842(3)	C1-O2	1.2855(16)
C1-O4	1.264(15)	C1-O1	1.2997(3)	C1-O1	1.2842(3)	C1-O2	1.2855(16)
mean	1.264		1.299		1.284		1.282
C2-O1	1.20(3)						
C2-O2	1.199(14)						
C2-O2	1.199(14)						
mean	1.20						
C1-C1	3.892(9)	C1-C1	4.0444(5)	C1-C1	4.0484(5)	C1-C1	2.877(4)
C1-C2	3.883(12)	C1-C1	4.0444(5)	C1-C1	4.0484(5)		
C1-C2	3.89(2)	C1-C1	4.0444(5)	C1-C1	4.0484(9)		
C2-C1	3.955(9)						

E.N. Maslen, V.A. Streltsov, and N.R. Streltsova, X-ray study of the electron density in calcite, $CaCO_3$. *Acta Crystallographica B* 49, 636-641 (1993).

Yu Ye, J.R. Smyth, and Paul Boni, Crystal structure and thermal expansion of aragonite-group carbonates by single-crystal X-ray diffraction. *American Mineralogist* 97, 707–712 (2012).

```
data_14593_publ

_audit_creation_method  "from EXP file using GSAS2CIF"
_audit_creation_date                   2015-10-24T08:35
_audit_author_name                     Bryan_Chakoumakos
_publ_section_title
;Empirically testing vaterite structural models using neutron
diffraction and thermal analysis
;

_publ_contact_author_name            Bryan_Chakoumakos
_publ_contact_author_email           chakoumakobc@ornl.gov
_publ_contact_author_address
;Quantum Condensed Matter Division
Oak Ridge National Laboratory, P.O. Box 2008, bldg 8600, Oak Ridge,
Tennessee 37831
;


loop_
_publ_author_name
'Chakoumakos, B.C.'
'Pracheil, B.M.'
'Koenigs, R.P.'
'Bruch, R.M.'
'Feygenson, M.'

_publ_author_address
;Quantum Condensed Matter Division
Oak Ridge National Laboratory, P.O. Box 2008, bldg 8600, Oak Ridge,
Tennessee 37831

Environmental Science Division
ak Ridge National Laboratory, P.O. Box 2008, bldg 1505, Oak Ridge,
Tennessee 37831

Wisconsin Department of Natural Resources, Oshkosh, Wisconsin 54901

Wisconsin Department of Natural Resources, Oshkosh, Wisconsin 54901

Forschungszentrum Julich, Julich Centre for Neutron Science,
Julich, North Rhine-Westphalia, Germany 52425
;

_publ_requested_journal         'Dalton Transactions'
_publ_section_abstract
;Otoliths, calcium carbonate (CaCO3) ear bones, are among the most
commonly
used age and growth structure of fishes. Most fish otoliths are
```

```
comprised of
the most dense CaCO3 polymorph,aragonite. Sturgeon otoliths, in
contrast,
have been characterized as the rare and structurally enigmatic
polymorph,
vaterite-a metastable polymorph of CaCO3. Vaterite is an important
material
ranging from biomedical to personal care applications although its
crystal
structure is highly debated. We therefore sought to characterize the
structure
and microstructure of sturgeon otoliths using thermal analysis and
neutron
powder diffraction, which is used non-destructively.  We found that
while
sturgeon otoliths We found that while sturgeon otoliths are primarily
composed
of vaterite, they also contain the denser CaCO3 polymorph, calcite.
Our neutron
data provide enhanced discrimination of the carbonate group compared
to x-ray
data,owing to the different relative neutron scattering lengths, and
thus offer
the opportunity to uniquely test the more than one dozen crystal
structural
models that have been proposed for vaterite.  Of those, space group
P6522
model, a = 7.1452(5) A, c = 25.354(4) A, V = 1121.0018A3 provided the
best fit
to the neutron powder diffraction data.
;
data_14593_phase1

_pd_block_id
2015-10-24T08:35|14593_phase1|Bryan_Chakoumakos||

_pd_char_particle_morphology    powdered_Lake_Sturgeon_otolith
_chemical_name_systematic       CaCO3
_exptl_crystal_colour           white
_exptl_crystal_description      powder
_pd_char_colour                 white
_cell_measurement_temperature   293(2)
_pd_spec_mounting               '3 mm diameter glass capillary'
_pd_spec_mount_mode             transmission
_pd_spec_shape                  cylinder
_diffrn_ambient_temperature     293
_diffrn_radiation_type          neutron
_diffrn_radiation_source         'Spallation Neutron Source ORNL'
_diffrn_measurement_device_type  'neutron powder diffractometer'
_diffrn_detector                 'He-3 linear-PSD tubes'
```

```
_pd_instr_location               'NOMAD diffractometer, SNS, ORNL
(USA)'
_pd_meas_scan_method             tof
_diffrn_measurement_method       'neutron time-event mode'
_pd_instr_dist_src/spec          19500
_pd_instr_dist_spec/detc         500 #distance for 154deg detector
bank
# detector distances increase to 3.0 meters for 7deg detector bank
_pd_meas_2theta_fixed            67 #data from 31,67,122deg detector
banks used

loop_
_diffrn_radiation_wavelength
3.10
1.35
0.85
# 6.2, 2.7, and 1.7 Angstrom bandwiths with center wavelengths of
3.10, 1.35,
# and 0.85 Angstrom, repsectivley

_computing_structure_refinement  'GSAS (Larson and von Dreele, 2000)'
_computing_molecular_graphics    'VESTA (Momma and Isumi, 2008)'

loop_
_atom_type_symbol
_atom_type_scat_length_neutron
_atom_type_scat_source
C 6.646 International_Tables_Vol_C
O 5.803 International_Tables_Vol_C
Ca 4.70 International_Tables_Vol_C
_refine_ls_shift/su_max           0.05
_refine_ls_shift/su_mean          0.01
_refine_ls_number_parameters      37
_refine_ls_goodness_of_fit_all    4.11
_refine_ls_number_restraints      6
_refine_ls_matrix_type            full
_cell_measurement_reflns_used    2676
_cell_measurement_theta_min      12.5
_cell_measurement_theta_max      67.5
_refine_ls_number_reflns         2676
_pd_proc_ls_prof_R_factor        0.0479
_pd_proc_ls_prof_wR_factor       0.0571

_reflns_limit_h_min                    0
_reflns_limit_h_max                    12
_reflns_limit_k_min                    0
_reflns_limit_k_max                    7
_reflns_limit_l_min                    -35
_reflns_limit_l_max                    53
_reflns_d_resolution_high              0.502
```

```
_reflns_d_resolution_low               1.860

_chemical_name_common           'vaterite, calcium carbonate'
_chemical_formula_sum           'C Ca O3'
_chemical_formula_moiety        'C O3, Ca'
_chemical_formula_weight        100.09
_pd_phase_name                  vaterite

_pd_phase_mass_%                51.99(5)
_diffrn_reflns_number           2239
_reflns_number_total            2239
_cell_length_a                  7.1443(4)
_cell_length_b                  7.1443
_cell_length_c                  25.350(4)
_cell_angle_alpha               90.0
_cell_angle_beta                90.0
_cell_angle_gamma               120.0
_cell_volume                    1120.5(2)
_exptl_crystal_density_diffrn   2.670
_symmetry_cell_setting          hexagonal
_symmetry_space_group_name_H-M  'P 65 2 2'
_symmetry_space_group_name_Hall 'p 65 2 (0 0 1)'
_cell_formula_units_Z           18
loop_ _symmetry_equiv_pos_site_id _symmetry_equiv_pos_as_xyz
       1 +x,+y,+z
       2 x-y,+x,+z+5/6
       3 -y,x-y,+z+2/3
       4 -x,-y,+z+1/2
       5 y-x,-x,+z+1/3
       6 +y,y-x,+z+1/6
       7 x-y,-y,-z
       8 +x,x-y,-z+5/6
       9 +y,+x,-z+2/3
      10 y-x,+y,-z+1/2
      11 -x,y-x,-z+1/3
      12 -y,-x,-z+1/6

# ATOMIC COORDINATES AND DISPLACEMENT PARAMETERS

loop_
      _atom_site_type_symbol
      _atom_site_label
      _atom_site_fract_x
      _atom_site_fract_y
      _atom_site_fract_z
      _atom_site_occupancy
      _atom_site_thermal_displace_type
      _atom_site_U_iso_or_equiv
      _atom_site_symmetry_multiplicity
C
```

```
C2      0.6909(16)   0.0          0.5          1.0        Uiso
0.027(4)      6
O
O1      0.8588(23)   0.0          0.5          1.0        Uiso
0.027(4)      6
O
O2      0.6025(22)  -0.0088(31)   0.5409(4)    1.0        Uiso
0.027(4)     12
C
C1      0.3752(15)   0.3267(20)   0.16580(35)  1.0        Uiso
0.0243(17)   12
O
O3      0.5558(22)   0.3367(28)   0.1714(5)    1.0        Uiso
0.0243(17)   12
O
O4      0.2699(23)   0.3265(34)   0.2059(4)    1.0        Uiso
0.0243(17)   12
O
O5      0.2998(20)   0.3170(28)   0.1201(4)    1.0        Uiso
0.0243(17)   12
Ca
Ca1     0.0076(17)  -0.0076(17)   0.08333      1.0        Uiso
0.0098(34)    6
Ca
Ca2     0.6810(19)   0.3190(19)   0.08333      1.0        Uiso
0.0098(34)    6
Ca
Ca3     0.3435(19)   0.6565(19)   0.08333      1.0        Uiso
0.014(8)      6

loop_
      _geom_bond_atom_site_label_1
      _geom_bond_atom_site_label_2
      _geom_bond_distance
      _geom_bond_site_symmetry_1
      _geom_bond_site_symmetry_2
      _geom_bond_publ_flag
  C2        O1                1.200(11)  .      1_555  N
  C2        O2                1.200(11)  .      1_555  N
  C2        O2                1.200(11)  .      7_556  N
  C2        Ca1               2.998(16)  .      4_655  N
  C2        Ca1               2.998(16)  .      5_655  N
  C2        Ca2                3.265(6)  .      4_655  N
  C2        Ca2                3.265(6)  .      5_665  N
  C2        C1                3.884(11)  .      9_545  N
  C2        C1                4.575(11)  .      9_555  N
  C2        C1                3.955(18)  .      9_655  N
  C2        C2                 4.767(5)  .      2_544  N
  C2        C2                 4.767(5)  .      6_665  N
  O1        C2                1.200(11)  .      1_555  N
```

O1	O2	2.078(19)	.	1_555	N
O1	O2	2.078(19)	.	7_556	N
O1	Ca1	2.307(11)	.	4_655	N
O1	Ca1	2.307(11)	.	5_655	N
O2	C2	1.200(11)	.	1_555	N
O2	O1	2.078(19)	.	1_555	N
O2	O2	2.078(19)	.	7_556	N
O2	Ca1	2.933(20)	.	4_655	N
O2	Ca2	2.383(16)	.	4_655	N
O2	Ca3	2.582(23)	.	4_665	N
C1	O3	1.264(7)	.	1_555	N
C1	O4	1.264(7)	.	1_555	N
C1	O5	1.264(7)	.	1_555	N
C1	Ca1	3.271(9)	.	1_555	N
C1	Ca2	3.045(17)	.	1_555	N
C1	Ca2	3.249(16)	.	6_565	N
C1	Ca3	3.241(20)	.	1_555	N
C1	Ca3	2.967(7)	.	6_555	N
C1	C1	3.892(7)	.	11_655	N
C1	C1	3.892(7)	.	11_665	N
O3	C1	1.264(7)	.	1_555	N
O3	Ca2	2.431(16)	.	1_555	N
O3	Ca3	2.154(13)	.	6_555	N
O4	C1	1.264(7)	.	1_555	N
O4	Ca1	2.510(26)	.	6_555	N
O4	Ca2	2.354(25)	.	6_565	N
O4	Ca3	3.025(10)	.	6_555	N
O5	C1	1.264(7)	.	1_555	N
O5	Ca1	2.401(15)	.	1_555	N
O5	Ca2	2.872(22)	.	1_555	N
O5	Ca3	2.468(27)	.	1_555	N
Ca1	C2	2.998(16)	.	3_544	N
Ca1	C2	2.998(16)	.	4_654	N
Ca1	O1	2.307(11)	.	3_544	N
Ca1	O1	2.307(11)	.	4_654	N
Ca1	O2	2.933(20)	.	4_654	N
Ca1	O2	2.933(20)	.	9_545	N
Ca1	C1	3.271(9)	.	1_555	N
Ca1	C1	3.271(9)	.	12_555	N
Ca1	O4	2.510(26)	.	2_554	N
Ca1	O4	2.510(26)	.	11_555	N
Ca1	O5	2.401(15)	.	1_555	N
Ca1	O5	2.401(15)	.	12_555	N
Ca1	Ca1	4.2261(8)	.	2_554	N
Ca1	Ca1	4.2261(8)	.	6_555	N
Ca1	Ca2	4.167(13)	.	1_445	N
Ca1	Ca2	4.041(25)	.	1_455	N
Ca1	Ca2	4.167(13)	.	1_555	N
Ca1	Ca3	4.109(15)	.	1_445	N
Ca1	Ca3	4.156(31)	.	1_545	N

```
  Ca1       Ca3              4.109(15)  .      1_555  N
  Ca2       C2                3.265(6)  .      3_654  N
  Ca2       C2                3.265(6)  .      4_654  N
  Ca2       O2               2.383(16)  .      4_654  N
  Ca2       O2               2.383(16)  .      9_655  N
  Ca2       C1               3.045(17)  .      1_555  N
  Ca2       C1               3.249(16)  .      2_654  N
  Ca2       C1               3.249(16)  .     11_655  N
  Ca2       C1               3.045(17)  .     12_665  N
  Ca2       O3               2.431(16)  .      1_555  N
  Ca2       O3               2.431(16)  .     12_665  N
  Ca2       O4               2.354(25)  .      2_654  N
  Ca2       O4               2.354(25)  .     11_655  N
  Ca2       O5               2.872(22)  .      1_555  N
  Ca2       O5               2.872(22)  .     12_665  N
  Ca2       Ca1              4.167(13)  .      1_555  N
  Ca2       Ca1              4.041(25)  .      1_655  N
  Ca2       Ca1              4.167(13)  .      1_665  N
  Ca2       Ca3              4.099(15)  .      1_545  N
  Ca2       Ca3              4.177(32)  .      1_555  N
  Ca2       Ca3              4.099(15)  .      1_655  N
  Ca2       Ca3              4.2280(9)  .      2_654  N
  Ca2       Ca3              4.2280(9)  .      6_555  N
  Ca3       O2               2.582(23)  .      4_664  N
  Ca3       O2               2.582(23)  .      9_555  N
  Ca3       C1               3.241(20)  .      1_555  N
  Ca3       C1                2.967(7)  .      2_554  N
  Ca3       C1                2.967(7)  .     11_665  N
  Ca3       C1               3.241(20)  .     12_665  N
  Ca3       O3               2.154(13)  .      2_554  N
  Ca3       O3               2.154(13)  .     11_665  N
  Ca3       O4               3.025(10)  .      2_554  N
  Ca3       O4               3.025(10)  .     11_665  N
  Ca3       O5               2.468(27)  .      1_555  N
  Ca3       O5               2.468(27)  .     12_665  N
  Ca3       Ca1              4.109(15)  .      1_555  N
  Ca3       Ca1              4.156(31)  .      1_565  N
  Ca3       Ca1              4.109(15)  .      1_665  N
  Ca3       Ca2              4.099(15)  .      1_455  N
  Ca3       Ca2              4.177(32)  .      1_555  N
  Ca3       Ca2              4.099(15)  .      1_565  N
  Ca3       Ca2              4.2280(9)  .      2_554  N
  Ca3       Ca2              4.2280(9)  .      6_565  N

loop_
      _geom_angle_atom_site_label_1
      _geom_angle_atom_site_label_2
      _geom_angle_atom_site_label_3
      _geom_angle
      _geom_angle_site_symmetry_1
```

```
      _geom_angle_site_symmetry_2
      _geom_angle_site_symmetry_3
      _geom_angle_publ_flag
  O1        C2        O2            120.000(4)     1_555  .
1_555  N
  O1        C2        O2            120.000(4)     1_555  .
7_556  N
  O2        C2        O2            119.999(8)     1_555  .
7_556  N
  C2        O1        Ca1             113.7(6)     1_555  .
4_655  N
  C2        O1        Ca1             113.7(6)     1_555  .
5_655  N
  Ca1       O1        Ca1            132.6(12)     4_655  .
5_655  N
  C2        O2        Ca2            128.3(12)     1_555  .
4_655  N
  C2        O2        Ca3            119.4(12)     1_555  .
4_665  N
  Ca2       O2        Ca3             111.2(6)     4_655  .
4_665  N
  O3        C1        O4            120.000(6)     1_555  .
1_555  N
  O3        C1        O5           120.0005(24)    1_555  .
1_555  N
  O4        C1        O5             120.1(14)     1_555  .
1_555  N
  C1        O3        Ca2             106.7(7)     1_555  .
1_555  N
  C1        O3        Ca3             118.1(6)     1_555  .
6_555  N
  Ca2       O3        Ca3             134.4(6)     1_555  .
6_555  N
  C1        O4        Ca1             122.7(9)     1_555  .
6_555  N
  C1        O4        Ca2            125.0(10)     1_555  .
6_565  N
  Ca1       O4        Ca2             112.3(5)     6_555  .
6_565  N
  C1        O5        Ca1             123.3(8)     1_555  .
1_555  N
  C1        O5        Ca3             116.8(8)     1_555  .
1_555  N
  Ca1       O5        Ca3             115.1(6)     1_555  .
1_555  N
  O1        Ca1       O1             140.5(13)     3_544  .
4_654  N
  O1        Ca1       O4              139.3(8)     3_544  .
2_554  N
  O1        Ca1       O4               79.1(6)     3_544  .
```

11_555 N
O1 Ca1 O5 82.7(5) 3_544 .
1_555 N
O1 Ca1 O5 100.6(4) 3_544 .
12_555 N
O1 Ca1 O4 79.1(6) 4_654 .
2_554 N
O1 Ca1 O4 139.3(8) 4_654 .
11_555 N
O1 Ca1 O5 100.6(4) 4_654 .
1_555 N
O1 Ca1 O5 82.7(5) 4_654 .
12_555 N
O4 Ca1 O4 65.2(8) 2_554 .
11_555 N
O4 Ca1 O5 79.9(6) 2_554 .
1_555 N
O4 Ca1 O5 92.0(6) 2_554 .
12_555 N
O4 Ca1 O5 92.0(6) 11_555 .
1_555 N
O4 Ca1 O5 79.9(6) 11_555 .
12_555 N
O5 Ca1 O5 170.4(12) 1_555 .
12_555 N
O2 Ca2 O2 172.1(11) 4_654 .
9_655 N
O2 Ca2 O3 107.4(6) 4_654 .
1_555 N
O2 Ca2 O3 75.6(5) 4_654 .
12_665 N
O2 Ca2 O4 91.0(6) 4_654 .
2_654 N
O2 Ca2 O4 82.5(6) 4_654 .
11_655 N
O2 Ca2 O3 75.6(5) 9_655 .
1_555 N
O2 Ca2 O3 107.4(6) 9_655 .
12_665 N
O2 Ca2 O4 82.5(6) 9_655 .
2_654 N
O2 Ca2 O4 91.0(6) 9_655 .
11_655 N
O3 Ca2 O3 137.3(12) 1_555 .
12_665 N
O3 Ca2 O4 141.7(9) 1_555 .
2_654 N
O3 Ca2 O4 79.1(4) 1_555 .
11_655 N
O3 Ca2 O4 79.1(4) 12_665 .

```
2_654  N
  O3        Ca2       O4                    141.7(9)     12_665  .
11_655  N
  O4        Ca2       O4                    70.1(10)      2_654  .
11_655  N
  O2        Ca3       O2                     88.8(8)      4_664  .
9_555  N
  O2        Ca3       O3                     77.1(7)      4_664  .
2_554  N
  O2        Ca3       O3                     97.3(6)      4_664  .
11_665  N
  O2        Ca3       O5                    177.3(7)      4_664  .
1_555  N
  O2        Ca3       O5                   93.49(27)      4_664  .
12_665  N
  O2        Ca3       O3                     97.3(6)      9_555  .
2_554  N
  O2        Ca3       O3                     77.1(7)      9_555  .
11_665  N
  O2        Ca3       O5                   93.49(27)      9_555  .
1_555  N
  O2        Ca3       O5                    177.3(7)      9_555  .
12_665  N
  O3        Ca3       O3                   172.2(14)      2_554  .
11_665  N
  O3        Ca3       O5                    101.1(7)      2_554  .
1_555  N
  O3        Ca3       O5                     84.7(5)      2_554  .
12_665  N
  O3        Ca3       O5                     84.7(5)     11_665  .
1_555  N
  O3        Ca3       O5                    101.1(7)     11_665  .
12_665  N
  O5        Ca3       O5                     84.3(8)      1_555  .
12_665  N


data_14593_phase2

_pd_block_id
2015-10-24T08:35|14593_phase2|Bryan_Chakoumakos||

_pd_char_particle_morphology    powdered_Lake_Sturgeon_otolith
_chemical_name_systematic       CaCO3
_exptl_crystal_colour           white
_exptl_crystal_description      powder
_pd_char_colour                 white
_cell_measurement_temperature   293(2)
_pd_spec_mounting               '3 mm diameter glass capillary'
_pd_spec_mount_mode             transmission
```

```
_pd_spec_shape                   cylinder
_diffrn_ambient_temperature      293
_diffrn_radiation_type           neutron
_diffrn_radiation_source          'Spallation Neutron Source ORNL'
_diffrn_measurement_device_type  'neutron powder diffractometer'
_diffrn_detector                  'He-3 linear-PSD tubes'
_pd_instr_location                'NOMAD diffractometer, SNS, ORNL
(USA)'
_pd_meas_scan_method              tof
_diffrn_measurement_method        'neutron time-event mode'
_pd_instr_dist_src/spec           19500
_pd_instr_dist_spec/detc          500 #distance for 154deg detector
bank
# detector distances increase to 3.0 meters for 7deg detector bank
_pd_meas_2theta_fixed             67 #data from 31,67,122deg detector
banks used

loop_
_diffrn_radiation_wavelength
3.10
1.35
0.85
# 6.2, 2.7, and 1.7 Angstrom bandwiths with center wavelengths of
3.10, 1.35,
# and 0.85 Angstrom, repsectivley

_computing_structure_refinement  'GSAS (Larson and von Dreele, 2000)'
_computing_molecular_graphics    'VESTA (Momma and Isumi, 2008)'

loop_
_atom_type_symbol
_atom_type_scat_length_neutron
_atom_type_scat_source
C 6.646 International_Tables_Vol_C
O 5.803 International_Tables_Vol_C
Ca 4.70 International_Tables_Vol_C
_refine_ls_shift/su_max            0.05
_refine_ls_shift/su_mean           0.01
_refine_ls_number_parameters       37
_refine_ls_goodness_of_fit_all     4.11
_refine_ls_number_restraints       6
_refine_ls_matrix_type             full
_cell_measurement_reflns_used     2676
_cell_measurement_theta_min       12.5
_cell_measurement_theta_max       67.5
_refine_ls_number_reflns          2676
_pd_proc_ls_prof_R_factor         0.0479
_pd_proc_ls_prof_wR_factor        0.0571

_reflns_limit_h_min                     0
```

```
_reflns_limit_h_max                     12
_reflns_limit_k_min                     0
_reflns_limit_k_max                     7
_reflns_limit_l_min                     -35
_reflns_limit_l_max                     53
_reflns_d_resolution_high               0.502
_reflns_d_resolution_low                1.860

_chemical_formula_sum             'C3 Ca3 O9'
_chemical_formula_weight          300.27
_chemical_formula_moiety          'C3 O9, 3(Ca)'
_pd_phase_name                    calcite
_pd_phase_mass_%                  48.01(5)
_diffrn_reflns_number             437
_reflns_number_total              437
_cell_length_a                          4.9856(6)
_cell_length_b                          4.9856
_cell_length_c                          17.0464(34)
_cell_angle_alpha                       90.0
_cell_angle_beta                        90.0
_cell_angle_gamma                       120.0
_cell_volume                            366.94(9)
_exptl_crystal_density_diffrn     2.718
_symmetry_cell_setting            trigonal
_symmetry_space_group_name_H-M    'R -3 c:h'
_symmetry_space_group_name_Hall   '-r 3 2"c'
_cell_formula_units_Z             2
loop_ _symmetry_equiv_pos_site_id _symmetry_equiv_pos_as_xyz
      1 +x,+y,+z
      2 -y,x-y,+z
      3 y-x,-x,+z
      4 y-x,+y,+z+1/2
      5 -y,-x,+z+1/2
      6 +x,x-y,+z+1/2
     -1 -x,-y,-z
     -2 +y,y-x,-z
     -3 x-y,+x,-z
     -4 x-y,-y,-z+1/2
     -5 +y,+x,-z+1/2
     -6 -x,y-x,-z+1/2
    101 +x+1/3,+y+2/3,+z+2/3
    102 -y+1/3,x-y+2/3,+z+2/3
    103 y-x+1/3,-x+2/3,+z+2/3
    104 y-x+1/3,+y+2/3,+z+1/6
    105 -y+1/3,-x+2/3,+z+1/6
    106 +x+1/3,x-y+2/3,+z+1/6
   -101 -x+2/3,-y+1/3,-z+1/3
   -102 +y+2/3,y-x+1/3,-z+1/3
   -103 x-y+2/3,+x+1/3,-z+1/3
   -104 x-y+2/3,-y+1/3,-z+5/6
```

```
    -105 +y+2/3,+x+1/3,-z+5/6
    -106 -x+2/3,y-x+1/3,-z+5/6
     201 +x+2/3,+y+1/3,+z+1/3
     202 -y+2/3,x-y+1/3,+z+1/3
     203 y-x+2/3,-x+1/3,+z+1/3
     204 y-x+2/3,+y+1/3,+z+5/6
     205 -y+2/3,-x+1/3,+z+5/6
     206 +x+2/3,x-y+1/3,+z+5/6
    -201 -x+1/3,-y+2/3,-z+2/3
    -202 +y+1/3,y-x+2/3,-z+2/3
    -203 x-y+1/3,+x+2/3,-z+2/3
    -204 x-y+1/3,-y+2/3,-z+1/6
    -205 +y+1/3,+x+2/3,-z+1/6
    -206 -x+1/3,y-x+2/3,-z+1/6

# ATOMIC COORDINATES AND DISPLACEMENT PARAMETERS


loop_
      _atom_site_type_symbol
      _atom_site_label
      _atom_site_fract_x
      _atom_site_fract_y
      _atom_site_fract_z
      _atom_site_occupancy
      _atom_site_thermal_displace_type
      _atom_site_U_iso_or_equiv
      _atom_site_symmetry_multiplicity
Ca
Ca1     0.0          0.0          0.0          1.0        Uiso
0.0044(24)    6
C
C1      0.0          0.0          0.25         1.0        Uiso
0.0140(23)    6
O
O1      0.2607(8)    0.0          0.25         1.0        Uiso
0.0132(11)   18

loop_
      _geom_bond_atom_site_label_1
      _geom_bond_atom_site_label_2
      _geom_bond_distance
      _geom_bond_site_symmetry_1
      _geom_bond_site_symmetry_2
      _geom_bond_publ_flag
  Ca1       Ca1             4.0444(5)  .    104_445  N
  Ca1       Ca1             4.0444(5)  .    104_545  N
  Ca1       Ca1             4.0444(5)  .    104_555  N
  Ca1       Ca1             4.0444(5)  .    204_444  N
  Ca1       Ca1             4.0444(5)  .    204_454  N
```

```
  Ca1       Ca1             4.0444(5)  .    204_554  N
  Ca1       C1            3.20989(32)  .    101_444  N
  Ca1       C1            3.20989(32)  .    101_544  N
  Ca1       C1            3.20989(32)  .    101_554  N
  Ca1       C1            3.20989(32)  .    204_444  N
  Ca1       C1            3.20989(32)  .    204_454  N
  Ca1       C1            3.20989(32)  .    204_554  N
  Ca1       O1             2.3479(21)  .    101_444  N
  Ca1       O1             2.3479(21)  .    102_544  N
  Ca1       O1             2.3479(21)  .    103_554  N
  Ca1       O1             2.3479(21)  .    204_554  N
  Ca1       O1             2.3479(21)  .    205_454  N
  Ca1       O1             2.3479(21)  .    206_444  N
  C1        Ca1           3.20989(32)  .    104_445  N
  C1        Ca1           3.20989(32)  .    104_545  N
  C1        Ca1           3.20989(32)  .    104_555  N
  C1        Ca1           3.20989(32)  .    201_445  N
  C1        Ca1           3.20989(32)  .    201_455  N
  C1        Ca1           3.20989(32)  .    201_555  N
  C1        O1               1.300(4)  .      1_555  N
  C1        O1               1.300(4)  .      2_555  N
  C1        O1               1.300(4)  .      3_555  N
  O1        Ca1            2.3479(21)  .    104_545  N
  O1        Ca1            2.3479(21)  .    201_555  N
  O1        C1               1.300(4)  .      1_555  N

loop_
      _geom_angle_atom_site_label_1
      _geom_angle_atom_site_label_2
      _geom_angle_atom_site_label_3
      _geom_angle
      _geom_angle_site_symmetry_1
      _geom_angle_site_symmetry_2
      _geom_angle_site_symmetry_3
      _geom_angle_publ_flag
  O1        Ca1       O1                 87.19(6)    101_444  .
102_544  N
  O1        Ca1       O1                 87.19(6)    101_444  .
103_554  N
  O1        Ca1       O1                    180.0    101_444  .
204_554  N
  O1        Ca1       O1                 92.81(6)    101_444  .
205_454  N
  O1        Ca1       O1                 92.81(6)    101_444  .
206_444  N
  O1        Ca1       O1                 87.19(6)    102_544  .
103_554  N
  O1        Ca1       O1                 92.81(6)    102_544  .
204_554  N
  O1        Ca1       O1                    180.0    102_544  .
```

```
205_454  N
  O1       Ca1      O1                 92.81(6)    102_544  .
206_444  N
  O1       Ca1      O1                 92.81(6)    103_554  .
204_554  N
  O1       Ca1      O1                 92.81(6)    103_554  .
205_454  N
  O1       Ca1      O1                    180.0    103_554  .
206_444  N
  O1       Ca1      O1                 87.19(6)    204_554  .
205_454  N
  O1       Ca1      O1                 87.19(6)    204_554  .
206_444  N
  O1       Ca1      O1                 87.19(6)    205_454  .
206_444  N
  O1       C1       O1                    120.0      1_555  .
2_555  N
  O1       C1       O1                    120.0      1_555  .
3_555  N
  O1       C1       O1                    120.0      2_555  .
3_555  N
#  Ca1      O1       Ca1                118.9(2)    104_543  .
201_555  N
#  Ca1      O1       C1                120.54(8)    104_543  .
1_555  N
  Ca1      O1       C1                120.54(8)    201_555  .
1_555  N
```
